# Role of Base Excision Repair Pathway in the Processing of Complex DNA Damage Generated by Oxidative Stress and Anticancer Drugs

**DOI:** 10.3389/fcell.2020.617884

**Published:** 2021-01-22

**Authors:** Yeldar Baiken, Damira Kanayeva, Sabira Taipakova, Regina Groisman, Alexander A. Ishchenko, Dinara Begimbetova, Bakhyt Matkarimov, Murat Saparbaev

**Affiliations:** ^1^School of Sciences and Humanities, Nazarbayev University, Nur-Sultan, Kazakhstan; ^2^National Laboratory Astana, Nazarbayev University, Nur-Sultan, Kazakhstan; ^3^School of Engineering and Digital Sciences, Nazarbayev University, Nur-Sultan, Kazakhstan; ^4^Department of Molecular Biology and Genetics, Faculty of Biology and Biotechnology, al-Farabi Kazakh National University, Almaty, Kazakhstan; ^5^Groupe ≪Mechanisms of DNA Repair and Carcinogenesis≫, Equipe Labellisée LIGUE 2016, CNRS UMR9019, Université Paris-Saclay, Gustave Roussy Cancer Campus, Villejuif, France

**Keywords:** inter-strand DNA crosslink, bulky DNA adduct, base excision repair, DNA glycosylase, nucleotide excision repair, Fanconi anemia

## Abstract

Chemical alterations in DNA induced by genotoxic factors can have a complex nature such as bulky DNA adducts, interstrand DNA cross-links (ICLs), and clustered DNA lesions (including double-strand breaks, DSB). Complex DNA damage (CDD) has a complex character/structure as compared to singular lesions like randomly distributed abasic sites, deaminated, alkylated, and oxidized DNA bases. CDD is thought to be critical since they are more challenging to repair than singular lesions. Although CDD naturally constitutes a relatively minor fraction of the overall DNA damage induced by free radicals, DNA cross-linking agents, and ionizing radiation, if left unrepaired, these lesions cause a number of serious consequences, such as gross chromosomal rearrangements and genome instability. If not tightly controlled, the repair of ICLs and clustered bi-stranded oxidized bases via DNA excision repair will either inhibit initial steps of repair or produce persistent chromosomal breaks and consequently be lethal for the cells. Biochemical and genetic evidences indicate that the removal of CDD requires concurrent involvement of a number of distinct DNA repair pathways including poly(ADP-ribose) polymerase (PARP)-mediated DNA strand break repair, base excision repair (BER), nucleotide incision repair (NIR), global genome and transcription coupled nucleotide excision repair (GG-NER and TC-NER, respectively), mismatch repair (MMR), homologous recombination (HR), non-homologous end joining (NHEJ), and translesion DNA synthesis (TLS) pathways. In this review, we describe the role of DNA glycosylase-mediated BER pathway in the removal of complex DNA lesions.

## Introduction

**Figure 1:**
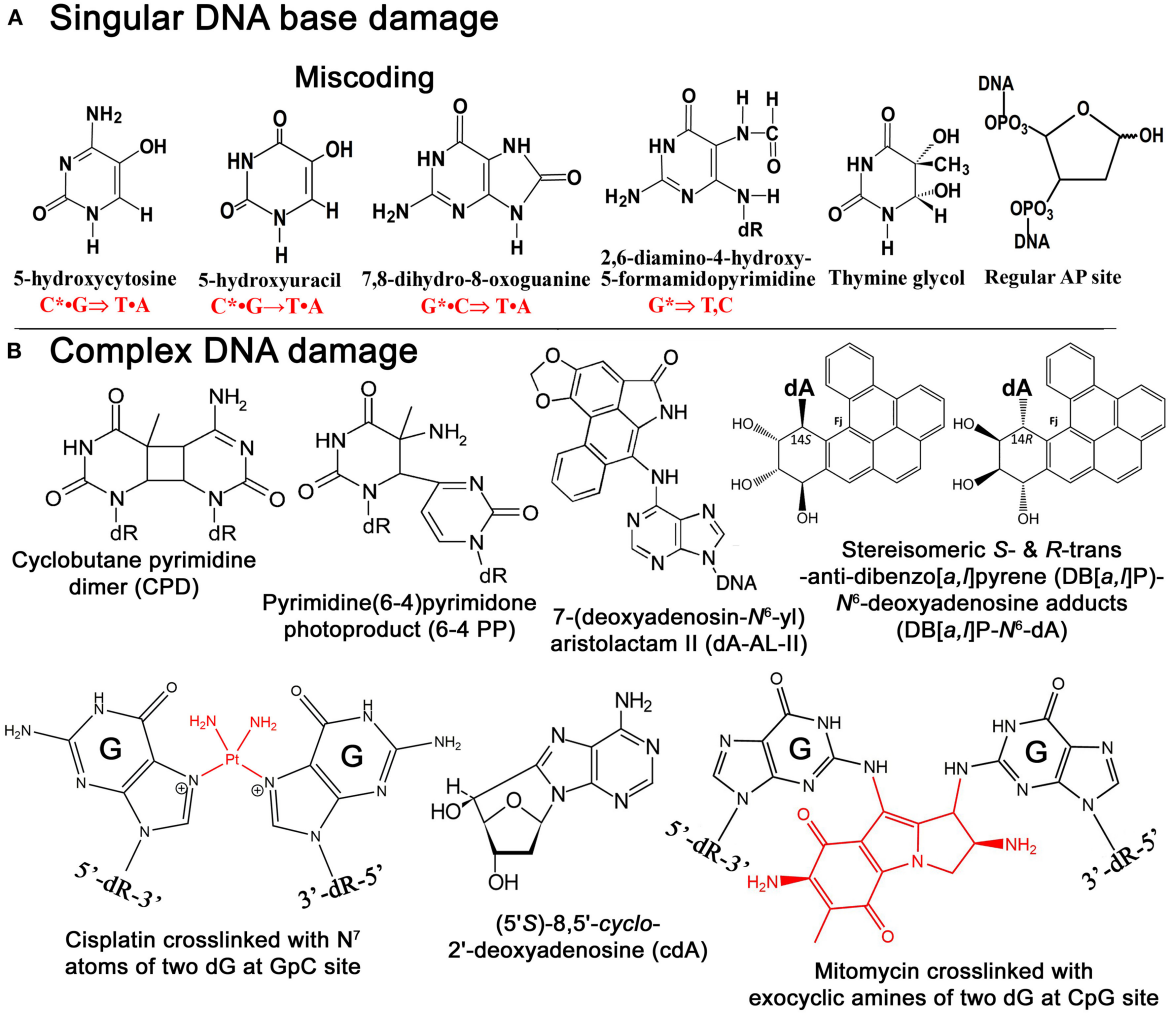
Chemical structures of singular and complex DNA base damage. **(A)** Singular non-bulky oxidative DNA base lesions. **(B)** Complex DNA lesions including cisplatin-ICL, mitomycin-ICL, and bulky repair-resistant DNA base lesions.

Endogenous oxidative stress and environmental factors induce multiple damage in cellular DNA, among them bulky DNA adducts, interstrand DNA cross-links (ICLs), and clustered DNA lesions (including double-strand breaks, DSBs), which are distinguished from singular lesions by their complex characters and structures (Deans and West, 2011; Yang et al., 2017; Mullins et al., 2019; Nickoloff et al., 2020) (Figures 1A,B). Complex DNA damage (CDD) is characterized by two important features: bulky character and the presence of more than one modification of one turn of the DNA helix. Although in general CDD constitutes a relatively minor share of the total DNA damage induced by chemo- and radiotherapy in treated cancer cells, they present daunting obstacles to DNA template scanning processes and if not repaired lead to cell death and gross chromosomal rearrangements. Among complex DNA lesions, ICLs are one of the most cytotoxic and difficult to repair, because they prevent DNA strand separation during DNA replication and transcription. To get insight into the biological roles of CDDs, it is important to comprehend the nature and mechanisms of their formation which often proceeds via interactions of DNA with endogenous reactive metabolites and various exogenous factors. Studies of the cellular defense mechanisms counteracting genotoxic effects of CDD revealed that the removal of complex lesions requires several distinct DNA repair pathways including global genome and transcription-coupled nucleotide excision repair (GG-NER and TC-NER, respectively), poly(ADP-ribose) polymerase (PARP)-mediated DNA strand break repair, base excision repair (BER), nucleotide incision repair (NIR), mismatch repair (MMR), non-homologous end joining (NHEJ), homologous recombination (HR), and translesion DNA synthesis (TLS) pathways. In this review, we attempt to enlighten the structural properties, mechanisms of formation of CDD, and role of recently discovered alternative DNA repair mechanisms. Due to the space constraints, the formation and repair of clustered DNA lesions will not be discussed in this review instead; we would recommend several excellent reviews on this topic (Cadet et al., 2012; Georgakilas et al., 2013; Sage and Shikazono, 2017; Mavragani et al., 2019).

## Complex DNA Damage—Nature, Formation, and Biological Role

### 8,5′-Cyclopurine-2′-deoxynucleosides

Reactive oxygen species (ROS) are commonly responsible for the formation of non-bulky DNA base damage in cellular DNA (Figure 1A). Majority of ROS-induced DNA lesions are eliminated in the DNA glycosylase-initiated BER pathway. Nevertheless, ROS can generate CDDs, which can strongly perturb DNA replication and transcription in mammalian cells. 8,5′-Cyclopurine-2′-deoxynucleosides (cPus) can be formed as a result of endogenous oxidative stress and also by ionizing radiation under anoxic conditions (Xu et al., 2014). Bulky cPus adducts including diastereomeric (5′*S*)- and (5′*R*)-8,5′-*cyclo*-2′-deoxyadenosine (cdA) and 8,5′-*cyclo*-2′-deoxyguanosine (cdG) are generated by hydroxyl radical attack at C5′ sugar by hydrogen atom abstraction resulting in the formation of a covalent bond between the C5′ and C8 positions of the purine nucleoside (Dirksen et al., 1988; Kuraoka et al., 2000; Brooks, 2008) (Figure 1B). These nucleotide modifications can be referred to as a tandem lesion in which both sugar and base moieties have been damaged within distance of single nucleotides (Kuraoka et al., 2000; Merecz and Karwowski, 2016). The additional covalent bond in cPu hinders cleavage of the glycosidic bond by DNA glycosylases, making these lesions substrates for the NER pathway (Brooks et al., 2000; Kuraoka et al., 2000; Kropachev et al., 2014). It was demonstrated that Cockayne syndrome (CS) complementation group A (CSA) protein, a DNA excision repair protein involved in the TC-NER sub-pathway, is required for the removal of 5′S-cdA adducts in DNA from primary skin cells (D'Errico et al., 2007). The GG-NER pathway can remove cdA adducts with a similar efficiency as that of UV-induced CPDs but displays higher activity in excising the R-diastereomers as compared to S conformations (Brooks et al., 2000; Kuraoka et al., 2000). Hence, high levels of 5′S-cdA diastereomer in the nonexposed control group of mice compared to analogous 5′R-cdA might be due to the more efficient removal of 5′R-cdA by the GG-NER pathway (Wang et al., 2012; Chatgilialoglu et al., 2019). Several studies suggested that 5′R-cdA and 5′R-cdG adducts in DNA are better NER substrates than the 5′S-cdA and 5′S-cdG lesions (Kropachev et al., 2014; Cai et al., 2015; Shafirovich et al., 2019). In agreement with this, cdG and cdA adducts accumulate in DNA of keratinocytes from NER-deficient xeroderma pigmentosum (XP) complementation group C (XPC) and CSA patients exposed to X-rays and potassium bromate (KBrO_3_) (D'Errico et al., 2006, 2007), as well as in organs of CSB^−/−^ knockout mice (Wang et al., 2012; Chatgilialoglu et al., 2019). Kinetic assays showed the involvement of TLS DNA polymerases including Pol η, Pol κ, Pol ι, and Pol ζ in the replicative bypass of cPu lesions in human cells (You et al., 2013). Interestingly, Xu et al. (2014) revealed a novel repair pathway for cPu lesions that results in trinucleotide repeat (TNR) deletion *via* a unique DNA polymerase β (Polβ) lesion bypass during DNA replication and BER.

### Aristolochic Acids and Their Association With Genotoxicity and Cancer Progression

Aristolochic acids (AAs) are a group of acids found in the flowering plant family *Aristolochiaceae* which are popular supplements in Chinese medicine. AAs are structurally diverse blends corresponding to nitrophenanthrene carboxylic acids composed of two main constituents: principally 8-methoxy-6-nitrophenanthro-(3,4-d)-1,3-dioxolo-5-carboxylic acid (AA-I, which is the most abundant one) and its 8-demethoxylated form (AA-II) (Dickman and Grollman, 2010). Consumption of AAs can lead to a rapid renal toxicity and, at certain levels, to liver (Ng et al., 2017; Lu et al., 2020) and kidney cancer (Vanherweghem et al., 1993; Debelle et al., 2008). Consumption of AAs-containing herbal remedies in South Asia remains a cause of serious concern for public health to date (Lu et al., 2020; Shan et al., 2020). Certain cases in Europe also have been registered in 1992; when several female patients in Belgium, after taking two Chinese herbs (*Stephania tetrandra* and *Magnolia officinalis*), as slimming treatment, suffered from rapidly progressive interstitial nephritis (Vanherweghem et al., 1993).

Although both AA-I and AA-II have analogous nephrotoxic and genotoxic effects in cells and animal models, carcinogenic mechanisms seem to be distinct (Schmeiser et al., 1986; Sato et al., 2004; Shibutani et al., 2007). It is noted that AA-I-induced nephrotoxicity has much severe consequences than the AA-II-induced one (Shibutani et al., 2007), even though AA-II can demonstrate greater genotoxicity and carcinogenic potential compared to AA-I (Shibutani et al., 2007; Xing et al., 2012). It was found that in the body aristolochic acids are activated by cellular nitroreductases, resulting in the formation of reactive intermediates that bind covalently to DNA to produce 7-(deoxyadenosin-*N*^6^-yl)aristolactam (dA-AL) and 7-(deoxyguanosin-*N*^2^-yl) aristolactam I (dG-AL) adducts (Bieler et al., 1997) (Figure 1B). The aristolactam-adenine adducts induce a unique mutational signature in tumors characterized by A•T → T•A transversions at dA residues located on the non-transcribed DNA strand (Moriya et al., 2011). This observation was further confirmed by Sidorenko et al., who demonstrated that dA-AL adducts are resistant to GG-NER, but can be efficiently removed by TC-NER (Sidorenko et al., 2012). Interestingly, the structural studies of damaged DNA have also revealed that the dA-AL adduct does not destabilize the DNA duplex, thus providing a structural basis for the mechanism of resistance of the bulky lesion to GG-NER (Lukin et al., 2012). Recently, it has been shown that translesion synthesis (TLS)-specific Y-family DNA polymerases, particularly Pol η, Pol ι, and Pol κ, are not required for the TLS across bulky dA-AL-I in mouse embryonic fibroblasts (MEFs), whereas Pol ζ, a B-family DNA polymerase, which is capable of bypassing the cyclobutane pyrimidine dimer (CPD), a common UV-induced lesion, catalyzes both insertion of dAMP and dTMP (Hashimoto et al., 2016).

### Di-Benzopyrene DNA Adducts Are Highly Mutagenic Lesions

Bulky DNA adducts generated by environmental carcinogens cause mutations that often drive malignant transformation of affected cells. As we described above, bulky DNA adducts are removed in the NER pathway; however, certain bulky DNA lesions that cause a minimal decrease, or an enhancement in the stabilities of the DNA duplex can be very resistant to the repair machinery. Benzo[a]pyrene (B[a]P) is a well-known representative of PAH carcinogens present in the environment (Luch, 2005). Primary sources of dibenzopyrenes in the environment are diesel and gasoline-fueled vehicle exhausts, tobacco smoke, other smoke sources such as grilling meat, coal gasification products, coal tar, and other substances produced by the incomplete combustion of organic matter (Luch, 2005; Bergvall and Westerholm, 2007; Kropachev et al., 2013). Cytochromes P450 1A1, 1A2, and 1B1 are responsible for the metabolic activation of PAHs by mono-oxygenation of hydrocarbons in cells to form epoxides (Luch et al., 1999; Straif et al., 2005; Sulc et al., 2016). Cellular metabolism of B[a]Ps produces a diol epoxide benzo[a]pyrene-7,8-dihydrodiol-9,10-oxide (BPDE), which can be hydrolyzed to BPDE tetrols and interact with guanines in DNA to form bulky B[*a*]P-*N*^2^-dG adducts and B[*a*]P-*N*^6^-dA adducts. B[a]Ps induces primarily guanine transversions (G•C → T•A).

Dibenzo[*a,l*]pyrene (DB[*a,l*]P), another PAH present in about ten-fold lower concentration in the environment, is 100 times more tumorigenic than B[*a*]P in rodent model systems (Cavalieri et al., 1991; Amin et al., 1995a,b; Prahalad et al., 1997; Luch, 2009; Zhang et al., 2011). The reactive diol epoxide intermediates of DB[*a,l*]P react with either *N*^6^-adenine or *N*^2^-guanine in DNA to form stable DB[*a,l*]P-*N*^6^-dA and DB[*a,l*]P-*N*^2^-dG adducts (Li et al., 1999a,b; Yagi et al., 2008) (Figure 1B). Remarkably, Kropachev et al. demonstrated that the *S* and *R* diastereoisomers of the DB[*a,l*]P-*N*^2^-dG adduct are 15 and 35 times, respectively, more susceptible to removal by the GG-NER machinery as compared to the stereo-chemically identical DB[*a,l*]P- *N*^6^-dA adduct (Kropachev et al., 2013). This observation suggests that the higher genotoxic activity of DB[*a,l*]P, as compared to B[*a*]P, might be due to the generation of repair-resistant and thus persistent DB[*a,l*]P-derived adenine adducts in exposed cells.

In summary, despite their bulky character, certain highly mutagenic DNA lesions can escape DNA damage surveillance and DNA excision repair. This repair-resistant DNA damage presents a challenge for the cell since it can persist in the genome and lead to the transcription and replication blockages and mutations. Nevertheless, these complex DNA lesions might be substrates for the TC-NER pathway, thus implying a possible existence of alternative DNA repair mechanisms that could remove damage in the non-transcribed part of genome.

### Interstrand DNA Cross-Link (ICL)—Formation, Nature, and Use in Anticancer Therapies

Chemical agents such as bifunctional alkylating agents, platinum compounds, antitumor antibiotics, and furanocoumarins when reacting with DNA bases can generate a covalent bond between nucleotides on the opposite strands of a DNA duplex resulting in the formation of interstrand DNA cross-links (ICLs). ICLs are highly cytotoxic DNA lesions that prevent DNA strand separation during DNA replication, transcription, and recombination. Indeed, DNA cross-linking agents, like mitomycin C, cis-diamminedichloroplatinum(II), and melphalan, are widely used against hyperplastic diseases, such as cancer.

The formation of ICLs can also be the result of endogenously occurring reactive aldehydes such as malondialdehyde, a natural product of lipid peroxidation of polyunsaturated fatty acids, and nitric oxide, which may induce diazotization of exocyclic amine groups of the bases (Scharer, 2005). It was estimated that a single unrepaired ICL could kill a bacterial or yeast cell, while about 40 unrepaired ICLs could kill a mammalian cell (Magana-Schwencke et al., 1982; Lawley and Phillips, 1996). There are several types of ICLs that were discovered and characterized very early in 1960s. Cisplatin or cis-diamminedichloroplatinum(II) (*cis*-DDP) reacts with the N^7^ atom of the deoxyguanosine residue forming initially one covalent bond with DNA then covalently binds to other neighboring guanine residues resulting in DNA cross-linking (Bancroft et al., 1990; Florea and Busselberg, 2011) (Figure 1B). Majority of *cis*-DDP-induced intra-strand DNA cross-links are between guanine residues in dGpG, dGpNpG, and between adenine and guanine in dApG sequences, and only a relatively minor portion of *cis*-DDP-induced ICLs are formed between two guanines in opposite strands at the dGpC context (Kartalou and Essigmann, 2001; Noll et al., 2006). The relative distribution of dGpG, dApG, and dGpNpG intra-strand cross-links, and dGpC ICLs, generated by *cis*-DDP is ~65, 25, 5–10, and 2–5% of total adducts, respectively (Noll et al., 2006; Jung and Lippard, 2007; Enoiu et al., 2012).

Nitrogen mustards contain a reactive N,N-bis-(2-chloroethyl)amine functional group which reacts with guanine and adenine at the N^7^ position with minor reaction at N^3^-dC, N^1^-dA, and *O*^6^-dG (Osborne et al., 1995; Florea-Wang et al., 2009; Rojsitthisak et al., 2011). Derivatives of nitrogen mustards induce various DNA base modifications, and only 5% among them represent DNA cross-links between the N^7^ position of two guanine bases on the opposite DNA strands (Kohn et al., 1966). Mustard derivatives such as chlorambucil, melphalan, cyclophosphamide, and bendamustine are widely used as chemotherapeutic agents that covalently cross-link two strands in the DNA duplex (Sunters et al., 1992; Chen et al., 2014).

Psoralens are a family of naturally occurring compounds known as the linear furanocoumarins linear furocoumarins found in leafy plants (mostly from *Apiaceae* and *Fabaceae*) (Scott et al., 1976). The hydrophobic and planar character of these molecules allows them to easily permeate the cell and then intercalate into the DNA duplex (Lopez-Martinez et al., 2016). Among several ICL-inducing agents, psoralens require UVA photoactivation following DNA intercalation to chemically react with DNA. 8-Methoxypsoralen (8-MOP), the most common form of psoralen frequently used for the treatments, is a planar, tricyclic compound that intercalates into the DNA duplex preferentially at 5′-TpA sites. Upon photoactivation, 8-MOP primarily photoalkylates DNA by cycloaddition to the 5,6-double bond of a thymine generating monoadducts (MA) with either the 4′,5′-double bond of the furan (MAf) or the 3,4-double bond of the pyrone (MAp) side of the psoralen (Cole, 1971). A unique property of psoralen photochemistry is that the absorption of a second photon by the MAf leads to formation of a pyrone side 5,6-double bond adduct with a flanking thymine in the complementary strand, thus generating an ICL (Johnston and Hearst, 1981).

Mitomycin C (MMC) is a product of *Streptomyces caespitosus*, a species of actinobacteria, commonly used as antineoplastic chemotherapeutic agent against bladder, breast, colorectal, head and neck, cervical, and non-small cell lung cancers, and adenocarcinoma of stomach and pancreas. MMC contains a variety of functional groups, including three-membered heterocyclic rings: aminobenzoquinone- and aziridine-ring systems, one amine and two methylene bridges (Tomasz, 1995). Inert by its nature, MMC can be activated via chemical or enzymatic reduction of quinone ring and subsequently it covalently links to two guanine residues in complementary DNA strands of 5′-CpG sequences through binding to the DNA minor groove (Noll et al., 2006; Fan and Peng, 2016) (Figure 1B). Specifically, after two-electron reduction of the quinone ring, MMC loses its methoxy group resulting in the formation of the hydroquinone intermediate. Tautomerization succeeded by the reaction with the N^2^-amino group of guanine results in monoadduct formation, whereas carbamoyl group elimination results in the formation of the highly reactive vinylogous hydroquinone methide intermediate, which subsequently alkylates the guanine on the opposite complementary DNA strand generating an ICL (Noll et al., 2006).

### Repair-Resistant Complex DNA Lesions Induced by Nontherapeutic Factors

Apart from commonly used chemotherapeutic drug agents, there are other known carcinogenic compounds such as tobacco-specific N-nitrosamines, fluoranthenes, naphthol and binol derivatives, aflatoxin B1, and acetaldehydes which can induce bulky DNA adducts and ICLs. Aflatoxin B1 (AFB1), a potent carcinogen produced by Aspergillus species, mainly found as food contaminant in animal farms and poultry production through the diet. AFB1 exposure of infant mice induces hepatocellular carcinoma after reaching adulthood (Vesselinovitch et al., 1972). AFB1 is activated by cytochrome P450 1A2 and 3A4 to the 8,9-epoxide consequently forming AFB1-N^7^-guanine adduct (Eaton and Gallagher, 1994). A relatively fast conversion rate of AFB1-N^7^-guanine adduct to the ring-opened, formamidopyrimidine form (FAPY-AFB1) is an essential step in promoting aflatoxin carcinogenesis. The FAPY-AFB1 adduct seems to be the most stable of all AFB1-DNA adducts and is quite resistant to DNA repair machinery (Martin and Garner, 1977; Eaton and Gallagher, 1994).

Acetaldehydes, abundant organic compounds, are highly reactive due to the electrophilic nature of their carbonyl carbon, causing a variety of cellular and chromosomal aberrations in human cells. The partial oxidation of ethanol in the liver by alcohol dehydrogenase produces acetaldehyde, which is converted into acetic acid by acetaldehyde dehydrogenases (ALDHs). Half of the population of Northeast Asian descent and about 5–10% of Northern European descent harbor a dominant mutation in the ALDH2 gene, which greatly reduces the enzyme activity and leads to acetaldehyde accumulation after alcohol consumption. Individuals with a mutant form of ALDH2 have a greater risk of liver damage and susceptibility to many types of cancer (Seitz and Meier, 2007). Acetaldehyde can generate ICL and protein-DNA cross-links. *N*^2^-Ethyl-2′-deoxyguanosine (*N*^2^-ethyl-dG) and 1, *N*^2^-propano-2'-deoxyguanosine (1, *N*^2^-PdG) are major DNA adducts caused by acetaldehydes. Several studies indicated that endogenous aldehydes are a significant source of genotoxicity in the human hematopoietic system and that the presence of the proficient Fanconi anemia (FA) pathway is essential to protect cells from DNA damage induced by these reactive compounds. Hematopoietic stem cells (HSCs) are a primary target for aldehyde-induced DNA damage, and FA-deficient patients suffer from bone marrow failure due to p53/p21-mediated cell death and senescence (Ceccaldi et al., 2012). In agreement with this model, it was shown that the HSCs in *Aldh2*^−/−^, *Fancd2*^−/−^ double KO mice accumulate more DNA damage than HSCs in either of the single knockout mice (Garaycoechea et al., 2012). Formaldehyde, another highly reactive compound, can be generated from abundant folic acid consumption but would be effectively handled by a two-tier protection glutathione-dependent formaldehyde dehydrogenase (ADH5)/FANCD2 mechanism; however, DNA damage may still happen and lead to cytotoxic effects. Individuals affected by FA lack the defense system against formaldehyde and might be prone to the toxic compound exposure from folate decomposition (Burgos-Barragan et al., 2017). On the other side, a new strategy based on the delivery of a formaldehyde in the form of folate derivatives along with ADH5 inhibition that could effectively kill breast cancer cells deficient for BRCA1 and BRCA2 has been recently proposed (Burgos-Barragan et al., 2017).

## Aberrant Repair of Complex DNA lesions

### Aberrant Repair of Bulky DNA Adducts

In general, DNA repair systems can discriminate between regular and modified bases. However, difficulties for the accurate discrimination between damaged and regular DNA strands do exist: DNA polymerase errors during replication and spontaneous conversion of 5-methylcytosine to thymine generate mismatched pairs between two regular bases. To thwart these mutagenic threats to genome integrity, cells have specific DNA repair mechanisms that can correct mismatched bases generated via spontaneous deamination or mis-incorporation during DNA replication. The mismatch-specific thymine- and adenine-DNA glycosylase (MBD4/TDG and MutY/MYH, respectively)-initiated BER and mismatch repair (MMR) pathway are able to eliminate normal DNA bases in mismatched DNA duplexes.

Evidences have accumulated that cellular response to DNA damage could lead to faulty DNA repair and contribute to age-related diseases such as cancer and brain disorder. In DNA repair-deficient cells and under certain circumstances, *E. coli* MutY and human MMR can act in an aberrant manner: MutY removes adenine (A) in a template strand opposite to the misincorporated 8-oxoguanine residue (Fowler et al., 2003), and MMR removes thymine opposite to *O*^6^-methylguanine (Hampson et al., 1997), leading to mutation and futile DNA repair cycles, respectively. These examples show that in the presence of unrepaired DNA lesions, the classic DNA repair mechanisms can act in an aberrant manner by targeting the nondamaged DNA strand and promote genome instability. Furthermore, the aberrant MMR and BER mechanisms acting upon oxidized guanine residues stimulate the trinucleotide expansion that underlies Huntington's disease, a severe hereditary neurodegenerative syndrome (Kovtun et al., 2007). The alkyl-purine DNA glycosylase (MPG/AAG/ANPG) initiates aberrant BER by removing regular purines from nondamaged DNA, and the increased level of MPG is associated with risk of lung cancer (Berdal et al., 1998; Leitner-Dagan et al., 2012). Actually, MMR is not capable of discriminating a particular strand in a DNA duplex harboring a mismatched base pair in absence of DNA replication (Pena-Diaz et al., 2012).

Previously, our laboratory showed that human TDG and MBD4 initiate aberrant repair by excising regular thymine (T) paired with a damaged adenine (A) residue in the DNA duplex (Talhaoui et al., 2014). TDG recognizes T in the nondamaged DNA strand opposite to 1, *N*^6^-ethenoadenine (εA), hypoxanthine (Hx), 8-oxoA, and even abasic site in the TpG/CpX sequence context, where X is a modified residue. MBD4 removes T only when it pairs with εA, but not with Hx and other modified adenine residues. *In vitro* reconstitution demonstrated that TDG can catalyze aberrant removal of T in the specific sequence context that leads to TpG, CpA → CpG mutations (Talhaoui et al., 2014). As we described above, the highly mutagenic aristolactam-adenine adducts (dA-AL-I and dA-AL-II) generated in DNA by metabolic activation of aristolochic acids (AAs) are associated with urothelial carcinomas of the upper urinary tract and chronic kidney disease (Grollman et al., 2007; Chen et al., 2012). The urothelial carcinomas associated with AA exposure are characterized by A → T transversions (73% of all substitutions) preferentially occurring on the non-transcribed DNA strand (Hoang et al., 2013; Rosenquist and Grollman, 2016). Remarkably, a very particular mutational signature characterized by frequent CAG → CTG transversions in the 5′-CpApG trinucleotide context has been observed in tumor cells from upper urinary tract carcinoma associated with AA exposure (Rosenquist and Grollman, 2016; Ng et al., 2017). Based on these observations, we hypothesize that TDG initiates aberrant removal of T opposite to dA-AL-I and dA-AL-II in the 5′-CpA^*^pG context and promotes A → T transversions in human cells via error-prone DNA repair synthesis. Thus, it is tempting to speculate that specific inhibition of TDG activity in human cells may prevent genotoxic effects of AA exposure.

MYH (MUTYH) is a human mismatch-specific adenine-DNA glycosylase homologous to the *E. coli* MutY protein; both repair proteins display very similar DNA substrate specificities. Mutations in the MUTYH gene are associated with the familial colorectal cancer in the absence of a germ-line mutation in the *APC* gene and confer a spontaneous mutator phenotype in human and mice cell lines (Al-Tassan et al., 2002). Noteworthy, Vrouwe et al., demonstrated that UV irradiation of the noncycling NER-deficient XP-C and XP-A human fibroblasts generated persistent single-strand DNA breaks 24 h after exposure, and activated ATR-dependent DNA damage response (Vrouwe et al., 2011). Intriguingly, the formation of single-strand DNA breaks and DNA repair synthesis initiated at damage sites in XP fibroblasts did not lead to removal of UV lesions in cellular DNA, which is the feature of aberrant repair. Recently, Mazouzi et al. (2017) showed that MUTYH promotes an increased UV sensitivity of XP cells. The authors suggested that in NER-deficient cells, MUTYH might inhibit a hypothetical, alternative NER-independent repair of UV-induced DNA damage. Here, we hypothesize that the human adenine-DNA glycosylase when acting upon the DNA duplex containing UV adducts targets adenines in the nondamaged complementary DNA strand. This in turn leads to aberrant futile repair of the nondamaged DNA strand and subsequent usage of the UV damaged DNA strand as a template for repair synthesis and ligation. Therefore, we speculate that the severe UV-sensitive phenotype of XP patients is due to a DNA glycosylase-initiated aberrant repair of UV-induced DNA lesions in human cells that would lead to persistent DNA strand breaks and mutations in both proliferating and nondividing cells.

### Aberrant Repair of Interstrand DNA Cross-Links

Similar to the alkyl-purine DNA glycosylases (AlkA and ANPG) which remove regular purines (Berdal et al., 1998), the NER machinery in bacterial and human cells can initiate futile DNA repair, during which regular oligonucleotide fragments are excised from undamaged DNA duplexes leading to the futile excision/re-synthesis cycles (Branum et al., 2001). Furthermore, mammalian NER machinery initiates futile repair when acting upon the DNA duplex, containing a single ICL, by excising a damage-free 22–28 mer oligomer near psoralen-induced DNA cross-link and generating a long gap (Bessho et al., 1997). Then, DNA repair synthesis fills the single-stranded gap and generates a non-ligatable nick without removing the ICL adduct (Mu et al., 2000). It is possible that in mammals the NER-mediated nonproductive repair of ICLs generates pro-apoptotic signals to eliminate cells containing nonrepaired or irreparable complex DNA lesions.

Cisplatin [*cis*-diamminedichloroplatinum(II)] is employed to treat various types of cancers, including lung, head and neck, ovarian, and other organs. Cisplatin interacts preferentially with guanine residues in DNA and generates mono-adducts and intra- and interstrand cross-links. Exposure of the cells to cisplatin triggers a strong DNA damage response signal, which often leads to the irreversible apoptosis. Studies of the roles of DNA glycosylases and other BER enzymes in the removal of ICL have generated conflicting results, suggesting that the involvement of a given DNA repair pathway strongly depends on cellular context and lesion structure. In cisplatin-ICL, the cytosine residues adjacent to the cross-linked guanines undergo extra-helical flipping which exposes them to water and subsequently stimulates spontaneous deamination and conversion of cytosines to uracils (Lukin and de Los Santos, 2006). A study performed by Kothandapani et al. (2011) showed that the inhibition of human major AP endonuclease 1, APE1, combined with the knockdown of uracil-DNA glycosylase (UNG) and DNA polymerase β (Polβ), makes cancer cells more resistant to cisplatin. *In vitro* reconstitution of the repair of cisplatin-ICL in synthetic oligonucleotide revealed that despite the presence of ICL, UNG excises neighboring uracil residues to generate AP sites, which are then cleaved by APE1, followed by the Polβ-catalyzed gap-filling DNA repair synthesis (Kothandapani et al., 2011). This futile BER adjacent to cisplatin ICL sites initiated by the DNA glycosylase-mediated excision generates persistent DNA strand breaks, which would interfere with the productive repair of ICLs and increase cisplatin cytotoxicity (Kothandapani and Patrick, 2013). Several APE1 inhibitors have been generated and explored, which can specifically target either AP site cleavage activity [methoxyamine and APE1 inhibitor compound III (API3)] or redox regulation function (E3330 and Gossypol/AT101) of human enzyme (reviewed in Laev et al., 2017). A recent study demonstrated that the combination of cisplatin treatment with inhibition of the redox function of APE1 by E3330 decreased migration and invasion of lung cancer cells (Manguinhas et al., 2020). Another study showed that APE1 redox inhibitors in combination with cisplatin inhibit proliferation of bladder cancer cells more efficiently than cisplatin alone (Fishel et al., 2019). Currently, specific inhibitors against BER proteins such as Polβ, PNKP, FEN1, Ligase IIIα, and PARP1/PARG have been developed either to sensitize several cancers or to form synthetic lethal partnerships with common cancer mutations (reviewed in Grundy and Parsons, 2020).

The study of repair of trioxsalen (psoralen)-induced ICLs in living cells by McNeill et al. (2013) demonstrated a possible involvement of human endonuclease VIII-like DNA glycosylase 1 (NEIL1) in the aberrant repair of complex DNA lesions. Using fluorescently tagged fusion proteins and laser micro-irradiation coupled with confocal microscopy, the authors demonstrated that NEIL1 accumulates at sites of digoxigenin-tagged trioxsalen (psoralen)-induced ICLs. In addition, NEIL1 binds to duplex DNA containing ICL without exhibiting a DNA glycosylase activity; apparently, this abortive interaction interferes with the recruitment of the XPC protein, a NER factor, and removal of ICLs. The authors proposed that NEIL1 specifically recognizes psoralen-ICLs, and this can obstruct the efficient removal of these lethal DNA lesions *in vivo* (McNeill et al., 2013).

## Removal of Bulky DNA Adducts in the Base Excision Repair Pathway

**Figure 2:**
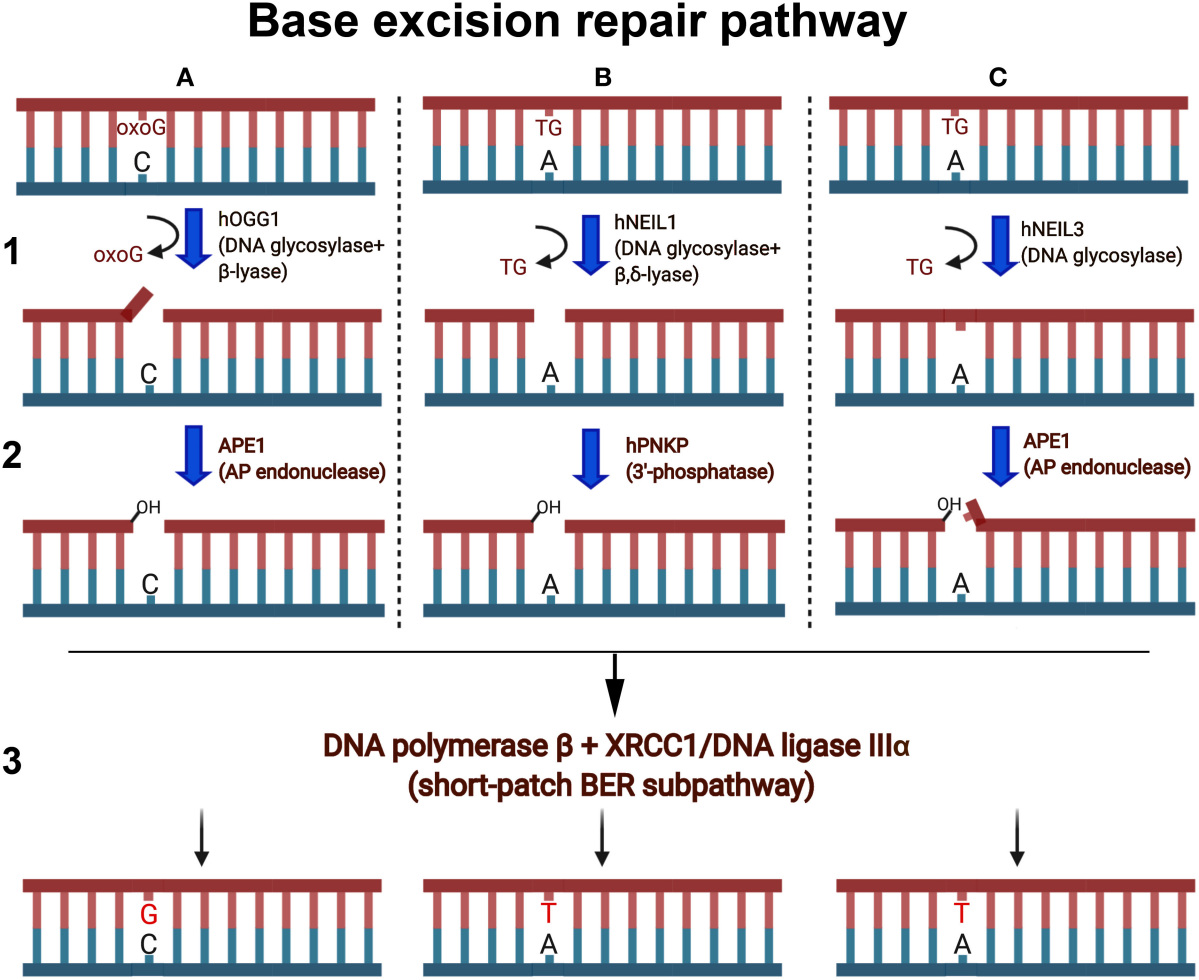
Schematic presentation of the short-patch BER pathways. Oxygen free radicals induce oxidized guanines (oxoG) and thymine glycol (TG) in duplex DNA. **(A1)** Human OGG1 DNA glycosylase excises oxoG base in duplex DNA and cleaves abasic site via β-elimination reaction and generates single-strand break containing 3′-terminal α,β-unsaturated aldehyde (3′-PUA) and a 5′-terminal phosphate. **(A2)** 3′-PUA is removed by major human AP endonuclease 1 (APE1) to generate 3′-OH termini. **(B1)** NEIL1 excises TG and cleaves the remaining abasic site via β,δ-elimination and generates one nucleotide gap flanked with 3′-P and 5′-P. **(B2)** 3′-Phosphate is removed by hPNKP to generate 3′-OH termini. **(C1)** NEIL3 cleaves the N-glycosydic bond, releasing the TG and generating an AP site. **(C2)** The resulting AP sites are incised at 5′ by APE1, which generates a single-strand break with 3′-OH and a 5′-blocking deoxyribosophosphate (dRP) group. **(A3,B3,C3)** Finally, DNA polymerase β inserts one nucleotide (dG or dT) and removes if necessary the 5′-dRp group by its dRp-lyase activity and the remaining single-strand break is then sealed by XRCC1/Ligase IIIα complex.

**Figure 3:**
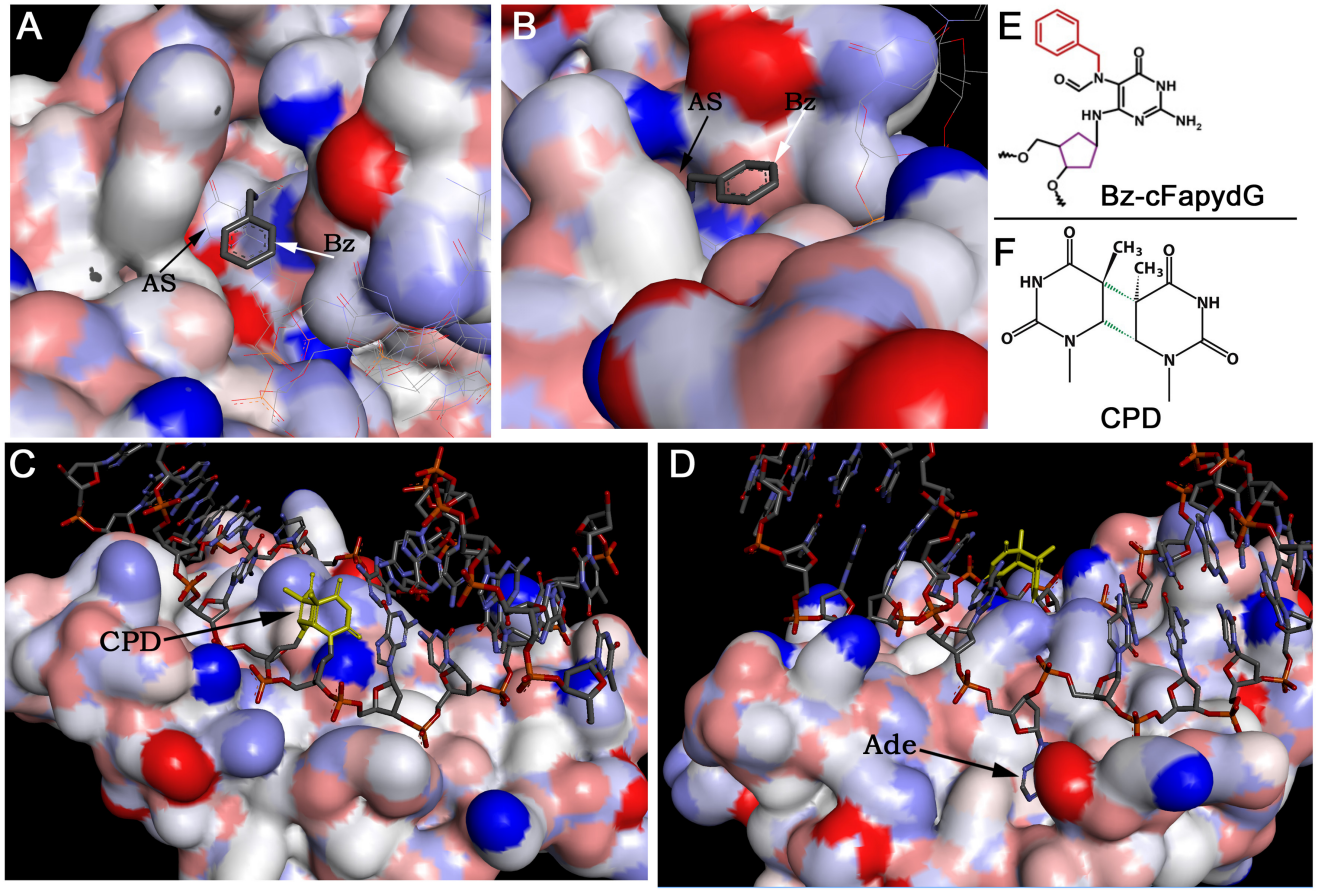
The crystal structures of DNA glycosylases in complex with DNA duplexes containing bulky DNA base modifications. **(A,B)** Focus view of the Fpg binding pocket with the extrahelical Bz-cFapydG adduct. The 3D views of DNA glycosylase/substrate interactions derived from the PDB entry 3C58 (Coste et al., 2008). The benzyl group of the N^7^-substituted cFapydG nucleoside is expelled from the binding pocket on the side of the flexible loop and thus allows the enzyme to accommodate the FapyG residue inside the active site pocket. **(C)** Focus view of the T4 endonuclease V catalytically active residues interacting with the phosphate backbones in the vicinity of the CPD. The 3D views of T4 Endo V/CPD interactions derived from the PDB entry 1vas (Vassylyev et al., 1995). **(D)** Focus view of the T4 endonuclease V-binding pocket with the extrahelical adenine residue. The adenine base complementary to 5′-CPD moiety is completely flipped out of the DNA duplex and trapped in a cavity on the protein surface. **(E)** Structure of the bulky N^7^-benzyl-cFapydG nucleoside (Bz-cFapydG). **(F)** Structure of the cyclobutane pyrimidine dimer (CPD). “AS” denotes active site pocket of Fpg, “Bz” denotes benzyl group at the N^7^ position of cFapydG, “CPD” denotes UV lesion, “Ade” denotes adenine residue in DNA.

DNA glycosylases and AP endonucleases recognize and remove a variety of small non-bulky DNA base damages that in general have little influence on thermodynamic stability of the DNA helix (Figure 2). DNA glycosylases bind and flip out of the duplex the damaged nucleotide and insert it into the active site pocket, to stabilize the DNA substrate conformation enzyme fills the void left in the helix by inserting amino acid residues (Klimasauskas et al., 1994; Stivers, 2004; Hitomi et al., 2007). In general, the active sites of DNA glycosylases are small to accommodate large bulky base modifications; nevertheless, some observations indicate that DNA glycosylases can recognize and remove bulky DNA adducts despite steric constraints to fit these base lesions into their active site pockets. Earlier, it was shown that several DNA glycosylases such as *E. coli* Fpg, phage T4 endonuclease V, and human NEIL1 can excise the imidazole ring opened form of guanine-C8-N-hydroxy-2-aminofluorene adduct, UV-induced cyclobutane dimer, and psoralen-thymine monoadduct, respectively (Boiteux et al., 1989; Vassylyev et al., 1995; Couve-Privat et al., 2007). Bacterial Fpg accommodates bulky *N*^7^-substituted FapydG derivatives of guanine lesion in its active site pocket by enabling the *N*^7^-bulky group to stay outside of the protein surface (Coste et al., 2008) (Figures 3A,B,E). Studies of the crystal structure of T4 endonuclease V in complex with cyclobutane pyrimidine dimer (CPD) DNA showed that the enzyme kinks the DNA duplex and flips out the complementary adenine base in the opposite strand out of the DNA base stack, thus avoiding accommodation of bulky CPD within the protein surface (Vassylyev et al., 1995) (Figures 3C,D,F). At present, it is unclear how NEIL1 can recognize large voluminous DNA lesions such as psoralen-thymine, protein-DNA cross-links, and aflatoxin-Fapy-deoxyguanosine adducts (AFB1-Fapy-dG); we hypothesize that similar to bacterial Fpg, the human DNA glycosylase avoids steric hindrance by the exclusion of a bulky group from its active site pocket (Couve-Privat et al., 2007; McKibbin et al., 2013; Vartanian et al., 2017).

A new family of bacterial DNA glycosylases that use a non-base-flipping mechanism to recognize bulky DNA base damage and a certain type of ICL has been described recently (Mullins et al., 2015a,b; Mullins et al., 2017b). A distinct DNA glycosylase superfamily including AlkC and AlkD from *Bacillus cereus* (Alseth et al., 2006) can excise positively charged alkylpurines without inserting them into their active site pocket (Mullins et al., 2015b; Shi et al., 2018). Due to the absence of specific contacts between protein and damaged nucleobase, and the interaction with the deoxyribose-phosphate backbone, AlkD is capable of excising bulky DNA adducts such as pyridyloxobutyl (POB) adducts, products of cigarette-smoke carcinogen nitrosamine ketone, and N3-yatakemycinyl-adenine (YTMA) generated by yatakemycin (YTM), an extremely cytotoxic alkylating product (Xu et al., 2012; Mullins et al., 2015b, 2017a).

Azinomycin B (AZB), a secondary metabolite produced by soil-dwell bacteria *Streptomyces sahachiroi* and *Streptomyces griseofuscus*, induces ICLs in the DNA duplex by nucleophilic addition at the N7 positions of purines (Wang et al., 2016). Previously, it was shown that the coordinated action of the classical NER machinery and homologous recombination (HR) can efficiently remove ICLs in bacterial cells (Cheng et al., 1991). However, AlkZ DNA glycosylase of *Streptomyces sahachiroi* can unhook AZB-induced ICLs in the DNA duplex, thus establishing a new alternative ICL repair mechanism in this bacterium. AlkZ resolves AZB-ICLs through the cleavage of the N-glycosidic bond on both sides of the complementary DNA strands, where resultant AP sites can be repaired by bacterial AP endonuclease, Endo IV. This observation supports the hypothesis that the BER pathway alone can remove a certain type of bulky DNA lesions and ICLs (Noll et al., 2006; Huang and Li, 2013; Mullins et al., 2019). Consequently, cells lacking AlkZ are highly sensitive to AZB-induced ICLs, supporting the physiological relevance of AlkZ DNA glycosylase activity in *S. sahachiroi* (Wang et al., 2016). Both AlkD and AlkZ are DNA glycosylases that are able to recognize bulky and cross-linked DNA bases and play an important role in a toxin resistance mechanism in bacterial populations.

Recently, YcaQ, a new *E. coli* cationic alkylpurine DNA glycosylase homologous to AlkZ, has been identified. YcaQ can excise a broad range of DNA base lesions, including ICLs induced by nitrogen mustard. These studies demonstrate that the DNA glycosylase-initiated BER pathway is an alternative ICL repair pathway in bacteria, which may provide insight for potential mechanisms implicated in drug resistance in cancer cells (Martin et al., 2017; Mullins et al., 2017b).

The UV damage endonuclease (UVDE) is a DNA endonuclease that recognizes and incises DNA 5′ next to cyclobutane pyrimidine dimers (CPDs) and 6-4 photoproducts (6-4PPs) (Bowman et al., 1994; Yajima et al., 1995). In addition, UVDE can cleave the DNA duplex containing AP sites, cisplatin intra-strand DNA cross-links, uracil, and dihydrouracil and removes 3′-blocking groups at single strand break termini (Avery et al., 1999; Kanno et al., 1999). This UV DNA damage endonuclease activity is present in various eukaryotic microorganisms, including fungi *Schizosaccharomyces pombe* and *Neurospora crassa*, prokaryotes such as the Gram-positive bacteria *Bacillus subtilis* and *Deinococcus radiodurans* and Gram-negative bacteria *Thermus thermophilus*, and thermophilic archaeon *Sulfolobus acidocaldarius*. Meulenbroek et al. (2013) have resolved the crystal structure of *Sulfolobus acidocaldarius* UVDE (SacUVDE) in a pre-catalytic complex with DNA duplex containing a 6-4 photoproduct in the absence of divalent metal cations. This 3D structure revealed a novel dual dinucleotide flip mechanism for recognition of bulky cross-linked dipyrimidines. Archaeon SacUVDE flips the two purines opposite to the damaged pyrimidine bases into a dipurine-specific pocket, whereas the damaged pyrimidines are also flipped into another cleft (Meulenbroek et al., 2013).

## Role of Nei-Like DNA Glycosylases in the Repair of ICLs

Mutations in genes responsible for counteracting ICL-mediated genotoxic effects can lead to Fanconi anemia (FA) disorder. FA is a recessive cancer-prone syndrome which is characterized with bone marrow failure and hypersensitivity to ICLs and, to a milder extension, to ionizing radiation and oxidative stress (Mace et al., 2005). Several lines of evidence indicate that in vertebrates, repair of ICLs is coupled to DNA replication and coordinated by the Fanconi anemia (FA) pathway. The stalled replication fork unhooks an ICL via dual incisions on both sides of the lesion by the scaffolding protein SLX4 and structure-specific endonuclease XPF/ERCC1, and this results in the generation of a double-strand break (DSB) (Hodskinson et al., 2014; Klein-Dowel et al., 2014). The unhooked ICL fragment swings free of the duplex exposing a single-stranded gap. Translesion synthesis (TLS)-specific DNA polymerases catalyze bypass of the gap and produce a three-stranded DNA repair intermediate that contains a short DNA fragment covalently attached to the duplex. It was proposed that this unhooked ICL fragment is removed in the classic NER pathway (Sancar et al., 2004; Cipak et al., 2006). However, work by Couve et al. (2009) revealed that the oxidative DNA glycosylase *E. coli* Nei and human NEIL1 excise with high efficiency the psoralen-induced bulky unhooked ICL oligomer within a three-stranded DNA structure. Three Nei-like (NEIL) DNA glycosylases are present in mammalian cells; these proteins show structural homology to the Fpg and Nei proteins of *Escherichia coli* and initiate the BER pathway to remove oxidized bases from DNA (Liu et al., 2013) (Figures 2B,C). Both NEIL1 and NEIL3 are cell cycle regulated with expression topping in the S phase and late S/G2, respectively, whereas NEIL2 is expressed throughout the cell cycle in a constitutive manner (Neurauter et al., 2012; Hegde et al., 2013). NEIL1 and NEIL2 contain a highly efficient β/δ-lyase activity, whereas NEIL3 exhibit a very weak β lyase function. All three mammalian Nei-like DNA glycosylases demonstrate an unusual preference for single-stranded DNA substrates and other open DNA conformations such as bubbles, loops, and recessed DNA duplexes generated during DNA replication and transcription. NEIL3 is perhaps the most intriguing of the three due, (i) to its large structure that includes an extended C-terminal domain containing additional zinc finger motifs, (ii) the replacement of the usual proline residue as the nucleophile with valine, and (iii) its restricted expression pattern in mammalian cells (Liu et al., 2013). The observation that Nei-like proteins from *E. coli* to human cells can excise bulky unhooked psoralen-induced ICLs via simple one-step hydrolysis of the glycosidic bond between cross-linked base and deoxyribose sugar provides an alternative heuristic solution for the removal of complex DNA lesions. Complete reconstitution of the repair of unhooked ICL demonstrated that this bulky adduct can be processed in a short-patch BER pathway (Figures 2B,C). Based on these data, a model for the mechanism of ICL repair in mammalian cells that implicates the DNA glycosylase activity of NEIL1 downstream of XPF/ERCC1 and TLS repair steps was proposed (Couve et al., 2009).

Recent progress in understanding the repair mechanism of ICLs in vertebrates has revealed the existence of a DNA strand incision-independent repair mechanism. *In vitro* reconstitution of the ICL repair using plasmid DNA and cell-free extracts from *Xenopus* eggs revealed the DNA replication-coupled repair of ICL, which results in the formation of an X-shaped DNA structure due to convergence of two replication forks on the lesion (Raschle et al., 2008). Interestingly, when only one fork was stalled at the ICL in egg extracts, no ICL repair was observed suggesting that the convergence of two forks is required for the removal of the blocking lesion (Zhang et al., 2015). At the same time, using mammalian cells and DNA combing, Huang et al. (2013) investigated the collision of replication forks with fluorescently marked psoralen ICLs to visualize the process. The results showed that in the S phase the majority of psoralen-induced ICLs are bypassed through a replication-traverse pathway to resume DNA replication on the other side without removal of the blocking lesions. Thus, the dual-fork convergence and replication fork traverse, and an X-shaped DNA structure is produced around the ICL (Zhang and Walter, 2014). Noteworthily, these remaining ICLs are removed after DNA replication and no DSB formation is observed (Huang et al., 2013). Subsequently, Semlow et al. (2016) have shown that NEIL3 from *Xenopus laevis* can excise ICLs induced by psoralen and abasic site in X-shaped dsDNA structures through a DNA strand incision-independent repair mechanism, implying that this repair function may be one of the many roles of NEIL3 in highly proliferating cells. Following work by Martin et al. (2017) demonstrated that the purified human NEIL3 can cleave psoralen-induced ICL in single-stranded, three-stranded, and four-stranded DNA structures to produce long unhooked DNA oligomers containing either an AP site or a psoralen-thymine monoadduct. Furthermore, *E. coli* Nei and human NEIL1, similar to NEIL3, excise a psoralen-induced four-stranded DNA substrate to generate two unhooked DNA duplexes but with a nick. Noteworthily, the unusual DNA substrate specificities of these highly evolutionary conserved Nei-like enzymes imply the occurrence of very long three- and four-stranded cross-linked DNA–DNA structures that may originate *in vivo* via completion of DNA replication and TLS bypass of an unhooked ICL. In this hypothetical model, Martin et al. proposed that FANCM catalyze replication fork traverse through an unhooked ICL, without incision-mediated unhooking; this may produce either three- or four-stranded DNA molecules with a duplex cross-linked to long ssDNA or dsDNA molecules. Finally, these putative three- or four-stranded DNA structures can be excised by the Nei-like DNA glycosylases in the BER pathway; this alternative mechanism avoids formation of highly genotoxic DSB.

Recently, Li et al. (2020) have demonstrated that the NEIL3-catalyzed repair of psoralen-induced ICLs is physiologically relevant in mammalian cells. The authors found that NEIL3 plays a major role in the repair of psoralen-induced ICLs in mammalian cells, which is non-epistatic with the Fanconi anemia (FA) pathway, whereas the FA pathway is primarily responsible for the repair of ICLs induced by anticancer drugs such as MMC and cisplatin and proceeds via generation of DSBs. Noteworthily, PARP1 recruits NEIL3 to psoralen-induced ICLs in rapid manner, and PARP1 inhibitors block NEIL3 repair (Li et al., 2020). NEIL3 specifically interacts with the RUVBL1/2 complex (RuvB-like AAA ATPase 1 and 2), which function in both NEIL3 and FA pathways of ICL repair. The RING-type E3 ubiquitin ligase TRAIP (TRAF-interacting protein), which is essential for cell proliferation and replication fork progression through ICL, requires the recruitment of NEIL3 and depletion of TRAIP switch ICL repair to the FA-dependent incision repair pathway. Similar to the RuvBL1/2 complex, TRAIP functions upstream of NEIL3 and FA pathways for ICL repair.

As we described above, acetaldehyde, an endogenous and alcohol-derived metabolite, produced upon alcohol consumption induces DNA cross-links and provokes bone marrow failure in FA patients. A new excision-independent repair pathway, which functions in addition to the FA system, to resolve acetaldehyde-induced ICLs has been identified recently (Hodskinson et al., 2020). This new repair mechanism, similar to NEIL3 and FA pathways, requires replication fork convergence but catalyzes direct DNA damage reversal which leads to the breakage of the acetaldehyde cross-link, without excision of the lesion. After ICL breakage, the repair of remaining acetaldehyde-guanine mono-adduct requires TLS DNA polymerases REV1 and Pol ζ resulting in a distinct mutational spectrum (Hodskinson et al., 2020).

It should be noted that despite structural similarities among various ICLs, the level of distortion of the DNA duplex and choice of DNA repair mechanism would strongly depend on the chemical nature of ICL. The incision-dependent Fanconi anemia-coordinated network which proceeds via generation of DSB is a preferred repair pathway for cisplatin and nitrogen mustard-induced ICLs (Cipak et al., 2006; Raschle et al., 2008). On the other hand, ICLs generated by photoactivated psoralen and abasic sites would be preferentially repaired via DNA glycosylase-mediated excision of ICL (Semlow et al., 2016; Martin et al., 2017; Imani Nejad et al., 2020). Noteworthily, mouse embryonic fibroblast (MEF) knockout for NEIL3 exhibit modest sensitivity to cisplatin (Rolseth et al., 2013), suggesting that some cisplatin-induced ICLs might be processed via the BER pathway, as a backup repair system for FA and NER. It should be noted that DNA glycosylase-dependent repair of ICLs seems to be more mutagenic as compared to the versatile Fanconi anemia system (Semlow et al., 2016).

## Concluding Remarks and Future Perspectives

Continuous damage of cellular DNA together with repair deficiency results in genome instability and cancer. Majority of DNA damage: non-bulky base modifications, abasic sites, and single-strand breaks with 3′-blocking groups are removed in DNA glycosylase and AP endonuclease-initiated BER and NIR pathways, respectively, without significant activation of DNA damage signaling pathways and cell cycle checkpoints, whereas complex DNA damage such as bulky DNA adducts and interstrand DNA cross-links, which constitute only a minor fraction of total DNA damage, presents a major challenge for DNA repair and cell fate. To counteract complex DNA lesions, the cell employs specific DNA damage signaling systems and several distinct DNA repair pathways acting in a highly coordinated manner. The chemical nature of complex DNA lesions and the extent of helix distortion their produce play important roles in DNA damage signaling, repair, and mutagenesis. Typically, complex DNA lesions are handled in the classical NER pathway, which can be coupled to transcription. Contrary to DNA glycosylases, NER machinery does not have specificity to a base modification *per se* but rather recognizes a DNA helix distortion, whereas DNA glycosylases are highly specific to the base modifications, because a damaged base should fit into their active site pocket. Intriguingly, certain types of DNA glycosylases are involved in the repair of bulky DNA adducts and ICLs, which cannot be accommodated inside the enzyme body. As a solution to this problem, T4 endonuclease V DNA glycosylase and UVDE endonuclease interact not with a UV pyrimidine dimer but with opposite adenine bases by inserting them into their specific pockets. The NER machinery cannot efficiently recognize bulky DNA adducts which do not distort DNA helix conformation and do not destabilize thermodynamic stability of the duplex (e.g., aristolactam-DNA adducts and di-benzopyrine-adenine adducts). In addition, the mammalian NER pathway is not efficient when acting upon ICLs, for the reason that both strands in duplex DNA are damaged and that the repair proteins cannot discriminate between two strands to excise the lesion (Mu et al., 2000), whereas Nei-like family DNA glycosylases recognize the adducted nucleobase but expel the bulky cross-linked moiety out of the active site pocket (Coste et al., 2008; Couve et al., 2009). NEIL1 and NEIL3 can excise ICL in both DNA strands in a sequential manner; importantly, NEIL3 does not cleave the DNA strand after base excision and thus does not generate toxic DSB (Semlow et al., 2016; Martin et al., 2017).

Mismatch-specific DNA glycosylases such as *E. coli* MutY and human TDG and MBD4 are prone to aberrant repair. In addition, cisplatin-induced ICLs can be processed in a futile uracil-DNA glycosylase-initiated BER, which exacerbates a cytotoxic effect of this drug. Thus, unrepaired bulky DNA adducts and ICLs might be processed by DNA glycosylases in an aberrant manner by removing nondamaged DNA bases, which are either opposite to modified DNA bases or in close proximity to an ICL. The question why aberrant DNA repair is evolutionarily conserved from *E. coli* to human remains pertinent. Plausible interpretations of this could be that (i) aberrant repair via stimulation of mutations in the specific regulatory regions of genome could play a capacitor role in evolution of warm-blood animals (Talhaoui et al., 2014) and (ii) aberrant removal of nondamaged bases prevents mutations since it triggers a strong cellular response, which in turn induces apoptosis or senescence in cells that accumulate unrepaired DNA lesions.

NEIL3 is a particular DNA glycosylase that possesses an extended C-terminal domain, containing three additional zinc finger-binding domains that specifically interact with ssDNA and ADP-ribose polymers (Chen et al., 2020). NEIL3 plays an important role in the repair of psoralen-induced ICLs in mammalian cells (Li et al., 2020). Nevertheless, NEIL3 DNA glycosylase-mediated repair of ICLs might be error-prone, as compared to the classic Fanconi anemia pathway, because of the generation of unrepaired AP sites in single-stranded DNA, which are protected from cleavage by the AP endonuclease (Ha et al., 2020) and can induce mutations (Semlow et al., 2016). Alternative DNA glycosylase-dependent repair of complex DNA lesions can be activated in cancer cells to promote genome instability and resistance to chemotherapy. Therefore, inhibition of DNA glycosylase activities in tumor cells offers a new strategy to combat anticancer therapy resistance.

## Author Contributions

YB, DK, AAI, DB, and MS wrote the nature of DNA damage part of the review. YB, ST, RG, BM, and MS wrote the DNA repair part of the review. YB, AAI, and MS prepared figures. All authors discussed and contributed to analysis of published literature and to writing the manuscript.

## Conflict of Interest

The authors declare that the research was conducted in the absence of any commercial or financial relationships that could be construed as a potential conflict of interest.
